# Development and evaluation of a novel training program to build study staff skills in equitable and inclusive engagement, recruitment, and retention of clinical research participants

**DOI:** 10.1017/cts.2022.456

**Published:** 2022-08-30

**Authors:** Jessica R. Cranfill, Stephanie A. Freel, Christine E. Deeter, Denise C. Snyder, Susanna Naggie, Nadine J. Barrett, Jamie N. Roberts

**Affiliations:** 1 Duke Office of Clinical Research, Duke University, Durham, NC, USA; 2 School of Medicine, Duke University, Durham, NC, USA; 3 Clinical and Translational Science Institute, Duke University, Durham, NC, USA; 4 Duke Cancer Institute, Duke University, Durham, NC, USA

**Keywords:** Research participation, community and stakeholder engagement, recruitment, retention, research training program, workforce development, clinical trials, health disparities, diversity, equity, inclusion, CTSA

## Abstract

**Background:**

Adequate equitable recruitment of underrepresented groups in clinical research and trials is a national problem and remains a daunting challenge to translating research discoveries into effective healthcare practices. Engagement, recruitment, and retention (ER&R) training programs for Clinical Research Professionals (CRPs) often focus on policies and regulations. Although some training on the importance of diversity and inclusion in clinical research participation has recently been developed, there remains a need for training that couples critical equity, diversity, and inclusion (EDI) concepts with skill development in effective recruitment and retention strategies, regulations, and best practices.

**Approach and methods:**

We developed the ER&R Certificate program as a holistic approach to provide Duke University CRPs the opportunity to build competency in gap areas and to increase comfort in championing equitable partnerships with clinical research participants. The thirteen core and elective courses include blended learning elements, such as e-learning and wiki journaling prompts, to facilitate meaningful discussions. Pre- and post-assessments administered to CRP program participants and their managers assessed program impact on CRP skills in ER&R tasks and comfort in equitable, diverse, and inclusive engagement of clinical research participants.

**Results and discussion:**

Results from the first two cohorts indicate that CRPs perceived growth in their own comfort with program learning objectives, especially those centered on participant partnership and EDI principles, and most managers witnessed growth in competence and responsibility for ER&R-related tasks. Results suggest value in offering CRPs robust training programs that integrate EDI and ER&R training.

## Background

The success of clinical research in improving public health depends on robust engagement, recruitment, and retention (ER&R) of participants that meet sample size requirements and represent the diversity of the population. Meeting enrollment goals in general can be challenging. As many as 19% of registered clinical trials are stopped early due to failed accrual [1], and as many as 86% do not achieve their accrual goals within their target timelines [2–4]. Within the context of health equity, this issue becomes even more salient as lack of participation among underrepresented race and ethnic groups compromises study outcomes and generalizability and can widen the gap in health disparities [5–8]. For example, underrepresented race and ethnic groups make up 36% of the US population and only account for < 12% of clinical research participants [9]. This critical negative impact is felt even more keenly when those carrying the greatest burden of a disease are not proportionately represented among research participants.

As Rodrigues-Torres *et al* have described, enrollment challenges fall into four general factor categories: study-related, participant-related, study team-related, and system-related [10]. Therefore, improving recruitment in clinical research will require multiple complex and multifactorial approaches. Thoughtfulness into systemic and individual factors, including stereotypes, systemic racism, and bias, and their influence on equitable recruitment of clinical research participants can significantly improve the overall health impact of interventions aiming to improve enrollment and retention. One key opportunity for intervention is training for the staff who engage research participants. Such training should build skills in specific areas such as Equity, Diversity, and Inclusion (EDI) and implicit bias, cultural humility, community engagement and outreach, and tailored communication that is inclusive, raises awareness, enhances trust, and incorporates perspectives of all potential participants [11].

Trainings on recruitment and retention competencies for Clinical Research Professionals (CRPs) are available [12–14]. However, many available offerings focus on rules, regulations, and policies regarding recruitment and informed consent. Recently, specific training programs have been created to address equity and diversity in clinical research participation [15–18]. These have demonstrated success in highlighting the necessity for enrolling underrepresented populations, although evidence of effectiveness to-date is somewhat limited [19,20]. Importantly, training programs for CRPs have not integrated regulatory and practical knowledge development with engagement and EDI principles, much less their impact on receuitment practices. Here we describe the development and implementation of a training program for CRPs that combines these critically interrelated concepts, which holistically we refer to as ER&R.

One aim of the Network Capacity Hub of Duke’s current Clinical and Translational Science Award (CTSA) is to provide recruitment training with an equity lens to investigators and staff. As a part of this aim, our curriculum is designed to build deeper skills in CRPs from across the Duke University Schools of Medicine and Nursing, including areas identified as critical educational gaps: personal internal biases and mitigation methods; knowledge of social marketing principles and their applicability to clinical research participation; attention to readability and health literacy needs; the value of adopting participant perspectives and building cultural humility; the importance of trust, trustworthiness and partnerships; sufficient budgeting for outreach; and community and stakeholder engagement [4,11].

The Duke ER&R Certificate Program is a training and skills-building program designed for staff-level CRPs, such as Clinical Research Coordinators (CRCs). The program’s purpose is to develop and expand ER&R competencies and to provide the tools and confidence necessary for staff to take proactive steps toward more inclusive ER&R practices in research conducted at Duke. This paper describes our efforts to develop, implement, and evaluate a blended instruction certificate program at Duke. We assess two cohorts of CRPs who have completed the certificate program.

## Methods

We began by establishing an interdisciplinary Steering Committee whose members planned, developed, implemented, and evaluated the ER&R program. Work was supported by both The Duke Clinical and Translational Science Institute (CTSI) and the Duke Office of Clinical Research (DOCR) and was funded by the Duke CTSA grant and the Duke University School of Medicine. The steering committee consisted of an expert (JR) in ER&R from the CTSI Recruitment Innovation Center (RIC), an expert (NJB) in health equity and inclusive research from the CTSI Equity in Research Core, an expert (JRC) in adult learning and instructional design from DOCR, and an expert (SAF) in workforce development and training from DOCR. We applied the Analysis, Design, Development, Implementation, and Evaluation (ADDIE) [21] instructional design model, which has been shown to produce effective training programs [21,22].

**Fig. 1.**
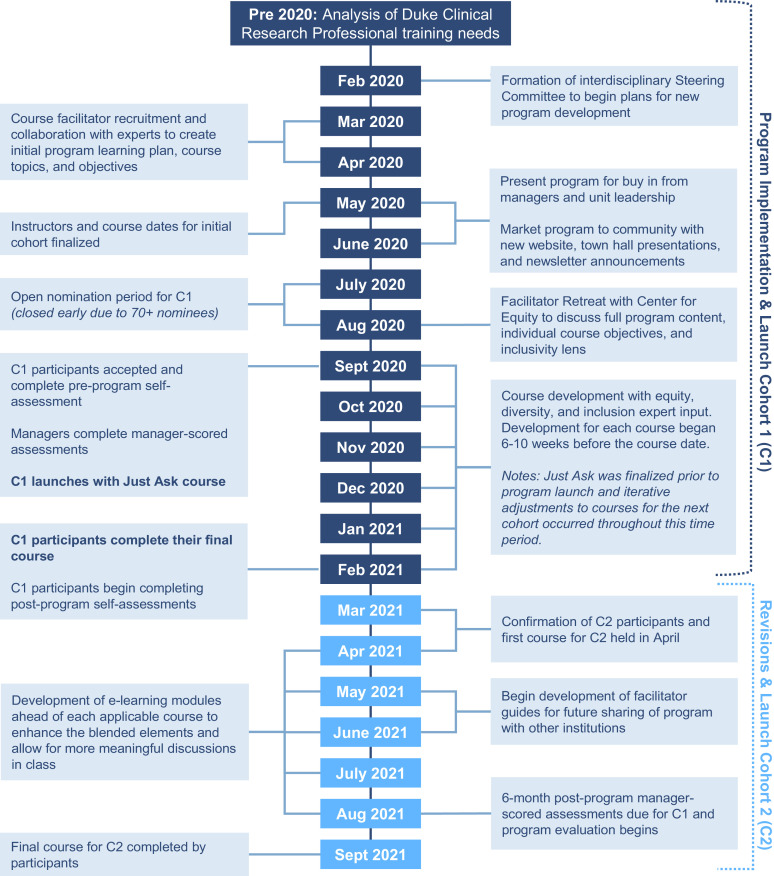
Program implementation and launch timeline for the first cohort (C1) and second cohort (C2) of the Engagement, Recruitment, and Retention program.

Program implementation took place over a period of 12 months, February 2020 to February 2021, as illustrated in Fig. 1. The goals for course and program design were to exemplify best practices in adult learning, incorporate EDI themes and application in each course, and prepare for program sustainability and ultimate sharing with other research institutions.

### Analysis Phase

We developed initial program goals and course objectives based on CRP training needs by engaging the Duke research community through a variety of leadership and CRP networks. The RIC team collated a list of potential training topics based on experience, a needs assessment survey, literature reviews, and consultations with the Duke research community. The steering committee identified and collaborated with subject matter experts (SME) from across the enterprise, gathering feedback on potential course content and program goals. Based on known challenges and literature cited above, the steering committee and SMEs agreed that a foundational focus of the program courses must be on EDI representation in research. From this Analysis phase, the following 4 key components were defined.

### Key Program Components

#### Expert instruction

Experts across Duke University helped develop the course objectives and content for their area of expertise, facilitate their course, and serve as resources for each topic. An expert in EDI (NJB) ensured facilitators were equipped with the tools, strategies, and framework needed to confidently shape and deliver content for each individual course with an equity lens.

#### Blended instruction design

The program includes e-learning, synchronous course sessions, and flipped classroom elements. Participants engage in meaningful discussion with facilitators and their peers after completing pre-learning materials.

#### Continuous engagement

Engaging course materials support ongoing learning, critical thinking, and application of concepts and skills throughout the program. The blended approach includes post-class journaling and continuous resource sharing among participants in a dedicated cohort Wiki space for each session.

#### Career development

Participants who complete the program receive a certificate that can be included in their portfolio for Duke CRP Tier Advancement opportunities [23].

### Design & Development Phases

The ER&R program consists of a series of 1- to 2-hour sessions, beginning with the *Just Ask: Equity and Diversity in Clinical Research* (NJB) course. *Just Ask* establishes an initial EDI mindset and provides participants with a foundation to begin identifying their own internal biases and ways to foster inclusive participant partnerships. The Just Ask instructor, along with other EDI experts from across Duke, contributed to the design of each individual course by guiding the development of learning objectives and framing course discussions and activities with an EDI lens. A full list of the courses and objectives for the first and second cohorts are outlined in Table 1. Those objectives that include course content related to EDI are indicated with a single asterisk. Additionally, course descriptions are publicly available on the ER&R program webpage.

**Table 1.** Core (C) and elective (E) courses and learning objectives (O#). The course title column lists program courses, blended learning elements, and any changes from cohort 1 to cohort 2. Double asterisks (**) in the course title column indicate changes or elements that were added for cohort 2 based on feedback and expansion of blended program design. The learning objectives column lists the objectives for each course. Objectives in **bold** were included in both cohort 1 and cohort 2. Objectives beginning with a single asterisk (*) are related to the program equity, diversity, and inclusion (EDI) lens given the content covered.

**Program Core Courses**	
Course Title	Learning Objectives
**C1:** Just Ask: Equity and Diversity in Clinical Research *Online Pre-Learning Module: Just Ask: Intro to Equity and Diversity in Clinical Research*	*O1: Define health disparity *O2: Define health equity *O3: Discuss what keeps diverse populations from accessing clinical research opportunities *O4: Describe your role in promoting diversity in clinical research *O5: Identify your own implicit biases *O6: Recognize how bias impacts recruitment and engagement in clinical research
**C2:** Clinical Research Recruitment, Regulations, Best Practices, and Tools **Cohort 1 held a virtual class, Cohort 2 moved to Online Pre-Requisite	**O1:** Discuss Duke policies related to recruitment and engagement **O2:** Recognize the process for obtaining Institutional Review Board (IRB) approval for recruitment plans and materials **O3:** Recall Duke Health branding requirements for materials and advertisements **O4:** Use Maestro Care tools to identify eligible participants * *O5: (Cohort 1 Only)* – Discuss recruitment plans and their feasibility
**C3:** Smarter to be Understood: Improving Readability **Online Pre-Learning Module: Readability Fundamentals + Participant Facing Engagement Materials	*O1: Apply readability foundations to produce materials participants can understand *O2: Recognize the importance of health literacy and readability in today’s scientific climate **O3:** Use available tools to perform a readability analysis of engagement materials **O4:** Recognize tools at Duke to develop lay-friendly materials **O5:** Develop a lay-friendly summary of your project to use in materials
**C4:** Retention: Challenges and Opportunities **Online Pre-Learning Module: Strategies to Support Retention of Clinical Research Participants	**O1:** Describe the importance of strong retention practices *O2: Set study expectations and explain them clearly to support retention *O3: Discuss strategies for relationship-building to support retention **O4:** Identify ways to assess continual participant interest in a study *O5: Recognize ways to discuss the importance of study continuation without coercion *O6: (Cohort 1 Only) –* Identify cues during recruitment that may lead to discontinued participation
**C5:** Using Social Marketing Principles to Design Your Engagement Strategy	**O1:** Define social marketing **O2:** Describe how evidence-based social marketing can be used to develop effective recruitment materials and strategies *O3: Identify strategies to recognize your audience and messages that will resonate with them *O4: (Cohort 1 Only) –* Recognize the importance of evidence-based marketing * *O5: (Cohort 1 Only) –* Identify ways to help marketing materials resonate with diverse audiences while being respectful of their perspective *O6: (Cohort 2 Only) – Discuss how formative research can help reach your audience *O7: (Cohort 2 Only)* – Describe the importance of tracking implementation and outcomes of a recruitment strategy
**C6:** Active Listening to Enhance Respect and Awareness of Participant Perspectives	**O1:** Define active listening *O2: Recognize the importance of active listening and how it can lead to both respectful and aware engagement and recruitment practices *O3: Recognize why listening is an important patient-centered engagement approach *O4: (Cohort 1 Only) –* Discuss concrete recruitment challenges with the study team and PI * *O5: (Cohort 1 Only) –* Identify strategies for being respectful during consent * *O6: (Cohort 2 Only)* – Identify strategies to build your capacity for hearing others * *O7: (Cohort 2 Only)* – Identify ways to shift your lens and consider other perspectives
**C7:** Building Trust and Partnerships **Moved from Electives to Core Courses for Cohort 2	*O1: Define trust and trustworthiness *O2: Recognize the importance of trust between study team and participants *O3: Discuss strategies for ensuring positive research interactions *O4: Identify strategies for building trust with the community at large

**Elective Courses (Participants Choose 3)**	
Course Title	Learning Objectives
**E1:** Community, Stakeholder, and Patient Engagement **Replaced with E4 and E8 for Cohort 2	* *O1: (Cohort 1 Only)* – Discuss engaging recruitment strategies and materials * *O2: (Cohort 1 Only)* – Identify patient engagement strategies in the design of your protocol * *O3: (Cohort 1 Only)* – Recognize components of a patient-centered study * *O4: (Cohort 1 Only)* – Identify and develop an engaging research question, outcomes, and endpoints * *O5: (Cohort 1 Only)* – Find tools and resources to help engage with patients and the community
**E2:** Social Media: Is it right for your research? **Online Pre-Learning Module: Using Social Media for Study Recruitment:Questions to Consider	**O1:** Identify questions to determine whether social media is right for a study **O2:** Discuss the use of social media kits to leverage existing Duke channels *O3: Recognize the basics of a social media plan including identifying goals, defining your audience, generating content, and measuring success **O4:** Recall social media common terms and tips **O5:** Find Duke guidelines, policies, procedures, and resources for marketing studies on social media *O6: (Cohort 1 Only)* – Discuss how to use Facebook advertising campaigns
**E3:** Telling The Story of Your Research	*O1: Recognize ways to communicate about research to a variety of audiences *O2: Discuss strategies for making research inviting rather than overwhelming **O3:** Identify tools to develop lay summaries of study results *O4: (Cohort 2 Only) –* Recognize opportunities to tell a story throughout different stages of a study
**E4: Community-Engaged Research Initiatives (CEnR) *Online Pre-Learning Module: Community & Stakeholder Engagement*	* *O1: (Cohort 2 Only) –* Define Community, Community Engagement and CEnR * *O2: (Cohort 2 Only) –* Describe the Principles of CEnR * *O3: (Cohort 2 Only) –* Describe why CEnR is important to addressing local priorities and improving well-being * *O4: (Cohort 2 Only) –* Describe how CEnR can address trust, increase diversity and inclusiveness and improve equity * *O5: (Cohort 2 Only) –* Discuss the spectrum of community engagement in research * *O6: (Cohort 2 Only) –* Find tools and resources to help you engage with the community
**E5:** ER&R on a Shoestring Budget	**O1:** Identify and plan for the real costs of engagement, recruitment, and retention **O2:** Recognize ways to plan for recruitment and engagement with a limited budget **O3:** Find tools and strategies for using your available recruitment budget * *O4: (Cohort 1 Only) –* Discuss the benefits of planning a budget for recruitment *O5: (Cohort 1 Only) –* Consider effort costs associated with robust recruitment strategies
**E6:** Remote Informed Consent: Design and Delivery Practices **Online Pre-Learning Module: Elements of Effective eConsent Design	**O1:** Recognize elements of effective eConsent design (cognitive load, multimedia, and interactivity) **O2:** Identifying ways to use remote consent platforms to your advantage by following principles of readability and human cognition *O3: Discuss social dynamics that may affect participant attitudes and preferences related to remote consent delivery **O4:** Identify tools and resources for eConsent
**E7:** 5Ts: A Framework to Support Inclusion of Older Adults in Research	*O1: Discuss case examples related to recruiting older adults *O2: Recognize why it is difficult to recruit older adults as a special population *O3: Define a framework for supporting inclusion of older adults in research *O4: Identify ways to include older adults in your studies
**E8: Principles and Best Practices of Stakeholder Engagement *Online Pre-Learning Module: Community & Stakeholder Engagement*	*O1: (Cohort 2 Only) – Recognize fundamental principles for stakeholder engagement *O2: (Cohort 2 Only) – Identify the right stakeholder engagement strategies for your study *O3: (Cohort 2 Only) – Describe how to identify stakeholders for a given study *O4: (Cohort 2 Only) – Discuss stakeholder engagement in the design and implementation of engagement, recruitment, and retention strategies and materials *O5: (Cohort 2 Only)* – Describe Duke tools and resources to help you identify and engage with stakeholders

Throughout implementation, the steering committee gathered feedback from participants and facilitators, making concomitant changes to the program structure and content. Notable changes to program structure occurred between the first cohort (C1) and second cohort (C2), including 1) requiring three electives rather than two; 2) splitting *E1: Community, Stakeholder, and Patient Engagement* into two separate courses; 3) adding five new online pre-learning modules; and 4) making *C7: Building Trust and Partnerships* a required core course rather than an elective. A full list of current program objectives is available in Supplemental Materials (A).

### Facilitator EDI Retreat

We recruited an interdisciplinary group of 21 facilitators from across Duke University. Prior to program launch, we hosted an all-facilitator retreat to ensure alignment with the EDI framework. The objective of the retreat was to discuss 1) how equity, diversity, and inclusion are integrated into each course’s topics and objectives; 2) the goal of the overall program; 3) how to address questions and facilitate discussions around EDI; and 4) appropriate language to use when discussing sensitive topics.

To further ensure the incorporation of intentional content tying in EDI principles and practices to each individual course concept, the steering committee was involved in the design of each course’s learning plan, subsequent materials, and activities. Learning plans for each course outlined: 1) content experts/facilitators, 2) method of instruction, 3) pre-learning requirements, 4) course description and objectives, and 5) post-class journaling prompts. Facilitator Guidebooks were created for each course to describe learning objectives, activities, and discussion prompts. This content organization strategy was used to ensure program sustainability and enable eventual sharing across institutions. Two example Course Learning Plans (B) and one Course Facilitator Guide (C) are available in the Supplemental Materials.

### Blended Learning Design

As described above, each course includes blended learning elements. The various pre-learning materials for the program include videos, journal articles, website reviews, and e-learning modules. The intention of most pre-learning experiences is to provide learners with an initial introduction to the course content, enabling them to attend the corresponding session with a foundational understanding of the topic. Other pre-learning materials prepare the participants for a specific activity or discussion that would occur during a session.

Both cohorts received an online introductory pre-learning module to supplement *Just Ask: Equity and Diversity in Clinical Research*. As of C2, six additional program courses include e-learning modules created (JRC) using the Articulate 360 Storyline and Rise development tools. To enhance information processing and recall, each module includes some combination of reading, visual and verbal elements (video, narration, and animation), interactive engagement, and assessment or practice [22,24].

A program Wiki provides a learning hub for materials and resource sharing [25]. Participants can access resources and share how they have used the various strategies they learned. The Wiki remains available after program completion for CRPs to review course materials and to share with colleagues. The Wiki houses the following for each cohort: 1) course title and description for each session, 2) resources (reading materials, videos, slides, web links, etc.), 3) pre-learning requirements including e-learning links, 4) post-class journaling prompts, 5) course evaluation link, 6) discussion space for comments and idea sharing. The post-class journaling prompts are intentionally designed to encourage program participants to think about the content covered with an inclusivity lens. With these prompts, we ask the program participants to reflect on various ways the content ties back to EDI recruitment and engagement of clinical research participants. Journaling prompt examples are included in the two Learning Plan samples in Supplemental Materials B. Course facilitators and members of the Steering Committee monitor ongoing conversations in the Wiki, providing additional information for consideration and thoughtful discourse.

### Implementation Phase

Initial implementation of the ER&R program occurred over a period of 12 months. Important steps to ensure successful implementation included gathering buy-in from Clinical Research Unit (CRU) managers to ensure their support of staff attending the program and marketing the program to the CRP community. This occurred via presentations and announcements provided first to CRU leadership and then to the full CRP community.

As shown in Fig. 1, the initial nomination period opened on July 6, 2020. CRU leadership and managers were encouraged to nominate CRPs who perform recruitment and retention functions. Nominations were closed one week earlier than anticipated due to overwhelming response. The steering committee reviewed all nominee applications and expected nominees to have sufficient experience to return to their units as ER&R mentors upon program completion. The program used a web-based data collection tool, REDCap, to house and track all forms and associated alerts for nomination, acceptance, elective selection, and evaluation [26].

Participants are required to complete one online pre-requisite, six core courses and attend at least three of seven elective courses to receive a certificate. Originally planned as in-person classes, the COVID-19 pandemic required a pivot to virtual sessions via Zoom. The first three cohorts were held biannually due to high demand for participation. Starting in 2022, Duke is offering the program once per year. Enrollment and completion tracking occur via the Duke Learning Management System (for e-learning and session attendance) and REDCap (for other elements) [26].

### Evaluation Phase

**Fig. 2.**
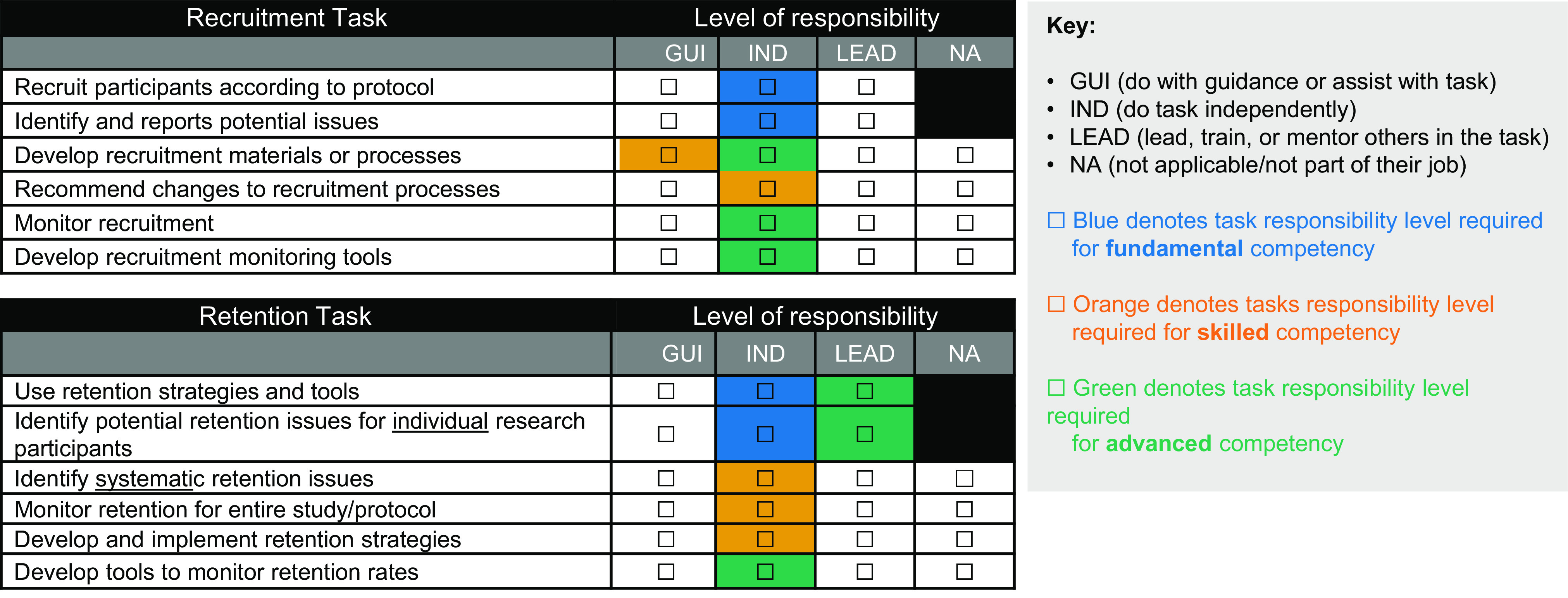
Recruitment and Retention tasks addressed in manager pre- and post-assessments of task responsibility.

Evaluations address the first three levels of the Kirkpatrick model of evaluation [27]: reaction, learning, and behavior. First, participants complete course evaluations following each session to rate success of each course at achieving learning objectives. Second, before and after program completion, participants complete identical self-assessments addressing their comfort with performing the session objectives for all core courses and their chosen electives on a six-point scale from very uncomfortable (1) to very comfortable (6). The self-assessments meet Kirkpatrick evaluation Level 2 and focus on self-perceived learning achieved during the program [27]. Third, managers complete assessments (Fig. 2 and Supplemental Materials D and E) before program start and six months after program completion to assess their employee’s level of competency in recruitment and retention-related tasks. These manager-scored assessments are existing validated tools used at Duke to measure competency achievement based on the JTFCR defined recruitment and retention competencies toward Tier Advancement [23]. This assessment meets Kirkpatrick evaluation Level 3, behavior [27].

We developed an interview guide (Supplemental Materials F) to collect additional information about participant experiences and takeaways from the program. Six students (3 from C1 and 3 from C2) participated in these interviews with an independent interviewer. This was a first step toward understanding participants’ perceptions of the program’s ability to promote integration of EDI principles into ER&R behavior.

## Results

C1 received 73 nominees within just a few days of opening the nomination process. With a planned cap of approximately 30 participants, we asked nominators from each CRU to prioritize two individuals for C1 and defer others to C2. All deferred individuals were guaranteed the opportunity to participate in C2. C1 graduated 32 CRPs, C2 graduated 25, and the third cohort graduated 23. Evaluation of the third and most recent cohort is still in process.

### Program Participants

A total of 59 CRPs, representing 18/23 Duke CRUs participated in C1 and C2, including three visiting participants from the University of North Carolina at Chapel Hill (UNC-CH) and one from Durham Technical Community College (DTCC), with a retention rate of 93% over the 6-month program. Table 2 displays information regarding program participant job titles, demographics, roles, and tenure in their position. Notably, almost 30% of program participants have worked in clinical research for seven or more years, indicating a perceived need for this type of training even for more seasoned staff. Program participants discussed the need for ongoing efforts to diversify the CRP workforce nationally, while ensuring access to training that incorporates an EDI lens to their work. It is encouraging that a relatively diverse group of CRPs were interested in the program and able to engage in rich discussions from a variety of perspectives.

**Table 2.** Participant information captured at nomination from cohort 1 and cohort 2. The table displays job title, length of time in clinical research, percentage of effort (time) spent on recruitment and retention tasks, and demographic information captured from 55 of 59 students, including race, ethnicity, sex, and age.

Participant Information and Demographics	
**Job Title**	**Count of Staff**
Clinical Research Coordinator Tier 1	16 (27%)
Clinical Research Coordinator Tier 2	10 (17%)
Clinical Research Coordinator Tier 3	4 (7%)
Clinical Research Coordinator, Senior	5 (8%)
Clinical Research Nurse Coordinator Tier 1	3 (5%)
Clinical Research Specialist, Senior	6 (10%)
Other	8 (13%)
Regulatory Coordinator Tier 3	1 (2%)
Research Program Leader Tier 1	5 (8%)
Research Program Leader Tier 2	1 (2%)
**Length of Time Worked in Clinical Research**	**Count of Staff**
< 1 year	2 (3%)
1 year - 3 years	18 (30%)
>3 years - 5 years	15 (25%)
>5 years - 7 years	8 (13%)
>7 years or more	17 (28%)
**Percentage Effort on Recruitment**	**Count of staff**
1 - 20%	9 (15%)
21 - 40%	21 (36%
41 - 60%	17 (29%)
61 - 80%	9 (15%)
81 - 100%	3 (5%)
**Percentage Effort on Retention**	**Count of Staff**
1 - 20%	13 (22%)
21 - 40%	29 (49%)
41 - 60%	12 (20%)
61 - 80%	3 (5%)
81 - 100%	2 (3%)
**Race**	**Count of Staff**
White	39 (71%)
Black or African American	12 (22%)
No Answer or Not Applicable	1 (2%)
Asian or Pacific Islander	2 (4%)
American Indian or Alaskan	1 (2%)
**Ethnicity**	**Count of Staff**
Hispanic/Latino	6 (11%)
Non-Hispanic/Latino	49 (89%)
**Sex**	**Count of Staff**
Female	52 (95%)
Male	3 (5%)
**Age (Years)**	**Count of Staff**
25–29	6 (11%)
30–34	11 (20%
35–39	13 (24%)
40–44	7 (13%)
45–49	3 (5%)
50–54	5 (9%)
55–59	8 (15%)
60–75	2 (4%)

### Self-Assessments

Self-assessments measured participant comfort with the course objectives listed in Table 1. Due to some changes in course offerings and objectives^1^ between C1 and C2, we analyzed objectives separately for C1 and C2 and then analyzed overlapping objectives between cohorts. For both C1 and C2, average comfort level grew from pre to post for every course objective. The average increase in comfort for each objective ranged from 7.6% to 42.4% (C1) and 8.5% to 52.6% (C2). The full data table is available in the Supplemental Materials (G).

The courses for C1 and C2 included 63 and 65 total learning objectives, respectively. Table 3 displays the ten objectives for each cohort with the highest increase in pre- to post-levels of comfort and indicates which objectives are directly related to EDI given the content covered. Five of the ten objectives with the greatest increase for C1 were in *E1: Community and Stakeholder Engagement*. Other notable growth for C1 (3 of top 10) occurred in the objectives for *E5: Shoestring Budget*. For C2, six of the top ten objectives were in *E4: Community-Engaged Research Initiatives* (2) and *E8: Stakeholder Engagement* (4) courses. Another notable increase in comfort for C2 occurred in the *C5: Social Marketing* (3 of 10).

**Table 3.** The 10 learning objectives with the highest percent increase in comfort according to cohort 1 and cohort 2 self-assessments. **Bolded rows** reflect community and stakeholder engagement-related objectives. Objectives beginning with a single asterisk (*) are related to the program’s equity, diversity, and inclusion (EDI) lens given the content covered

Course Learning Objectives with Highest Percentage (%) Increase in Comfort					
Cohort	Course Topic	Objective	Pre_Mean	Post_Mean	% increase
Cohort 1 (C1)	**Community, Stakeholder, and Patient Engagement**	*Find tools and resources to engage with participants and the community	**2.8**	**4.9**	**42.4%**
	**Community, Stakeholder, and Patient Engagement**	*Identify community and stakeholder engagement strategies in the design of your protocol	**3.2**	**4.9**	**35.6%**
	**Community, Stakeholder, and Patient Engagement**	*Discuss engaging recruitment strategies and materials	**3.3**	**5.0**	**33.3%**
	Shoestring Budget	Consider effort costs associated with robust recruitment strategies	3.1	4.6	33.3%
	Shoestring Budget	Identify and plan for the real costs of engagement, recruitment, and retention	3.0	4.5	32.8%
	Shoestring Budget	Discuss the benefits of planning a budget for recruitment	3.5	5.1	31.2%
	Social Media	Develop plans to leverage existing Duke social media channels	3.3	4.7	30.3%
	**Community, Stakeholder, and Patient Engagement**	*Recognize the components of a patient-centered research study	**3.6**	**5.1**	**29.5%**
	Social Marketing	*Identify ways to develop inclusive marketing materials that resonate with and respect diverse perspectives	3.6	5.1	29.0%
	**Community, Stakeholder, and Patient Engagement**	**Identify and develop an engaging research question, outcomes, and endpoints**	**3.4**	**4.8**	**28.1%**
Cohort 2 (C2)	**Community-Engaged Research (CEnR)**	*Describe the principles of CEnR	**2.5**	**5.2**	**52.6%**
	**Principles of Stakeholder Engagement**	**Describe Duke tools and resources to help you identify and engage with stakeholders**	**2.6**	**5.2**	**50.0%**
	**Community-Engaged Research**	*Define Community Engagement and Community-Engaged Research	**3.0**	**5.4**	**44.1%**
	**Principles of Stakeholder Engagement**	*Recognize the fundamental principles and best practices in stakeholder engagement for clinical research	**2.6**	**4.6**	**43.5%**
	**Principles of Stakeholder Engagement**	*Identify the right stakeholder engagement strategies for your study	**2.6**	**4.6**	**43.5%**
	Social Marketing	Describing how evidence-based social marketing can be used to develop engagement and recruitment material and strategies	2.8	4.9	43.0%
	**Principles of Stakeholder Engagement**	*Describe how to identify stakeholders for a given study	**3.2**	**5.6**	**42.9%**
	Social Marketing	*Discuss how formative research can help reach your audience	2.8	4.8	41.5%
	Social Media	*Recognize the basics of a social media advertisement plan	3.2	5.3	39.7%
	Social Marketing	Define social marketing	3.0	5.0	39.6%

As indicated in Table 1, over half of all individual course objectives (35/63 for C1 and 39/65 for C2) are related to EDI given the content covered in class. For C2, all but three EDI-related objectives had an average rating of 5 (comfortable) or higher after program completion. Notably, the C2 objectives with an average rating of less than 5 post-program were still rated high, between 4.6 and 4.9, and fall into the top 10 objectives with the most average growth from pre to post in Table 3. Similarly, for C1, only 4/35 EDI-related objectives had an average comfort rating of less than 5 (4.6–4.9) post-program. As shown in Table 3, two of these C1 objectives were the objectives with the most growth from pre to post for C1. Those corresponding C1 courses (*E3: Telling the Story, E6: Remote Consent, E1: Community and Stakeholder Engagement*) were updated significantly for C2.^a^

**Fig. 3.**
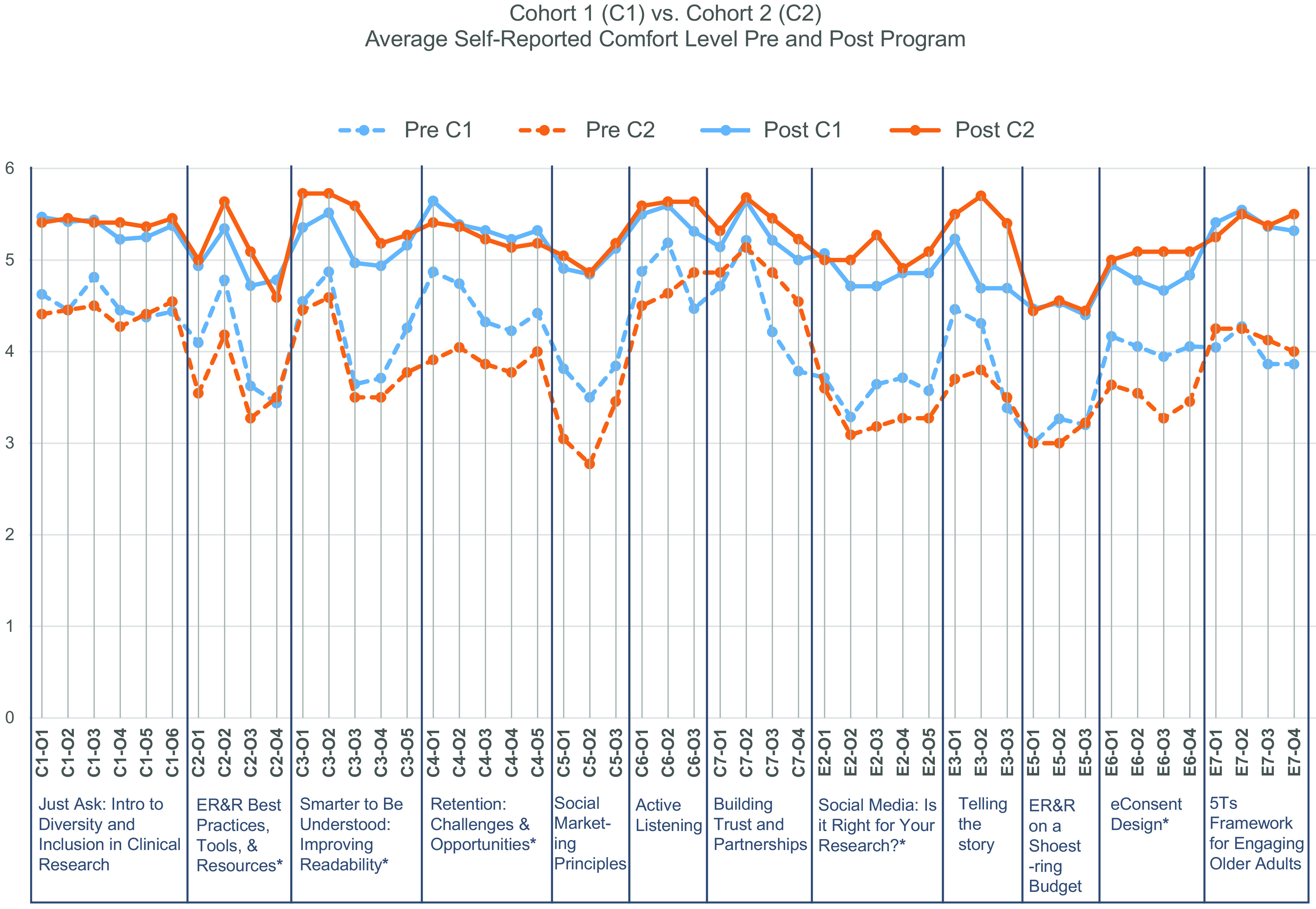
Average comfort level (very uncomfortable (1) to very comfortable (6)) with overlapping course objectives before and after program completion. Table 1 outlines the specific course objectives that map to the codes in this figure (e.g. C1-01 = Core 1, Objective 1 and E2-01 = Elective 2, Objective 1). A single asterisk (*) indicates courses where an additional e-learning module was added as pre-learning prior to holding the course for Cohort 2 (C2).

Fig. 3 shows mean comfort level pre- and post-program from 1 (very uncomfortable) to 6 (very comfortable) for course objectives that overlapped (stayed the same) between C1 and C2. This figure does not include data from the community-engaged research or stakeholder engagement-related courses, which had significantly different course offerings and objectives between cohorts.

For 39 (79.6%) of the 49 overlapping learning objectives, C2 showed more growth than C1 in average comfort level from pre to post. The higher comfort post-program and/or greater growth for C2 might be attributed to the addition of more blended elements for the second cohort, including creation of online pre-learning modules and additional discussion time in class. Every course that added a blended learning element for C2 showed greater overall comfort growth over C1. This difference between cohorts could also be attributed to the selection process for C1 and C2. C1 participants were hand selected from a list of 74 nominees by leadership in each unit as most likely to return to the unit as a resource upon program completion, possibly indicating a higher overall baseline comfort level.

For both cohorts, the objectives with the lowest pre-program comfort ratings (3 to 3.6 for C1 and 2.8 to 3.3 for C2) were in the *E5: ER&R on a Shoestring Budget*, *E2: Social Media*, and *C5: Social Marketing* courses. The objectives with the highest pre-program comfort (4.6 to 5.2 for C1 and 4.5 to 5.1 for C2) were in the *C7: Building Trust* and *C6: Active Listening* courses. There were notable differences between C1 and C2 percent increases in average comfort level for *E3: Telling the Story* and *E6: eConsent Design*, with C2 being less comfortable with the associated objectives pre-program and more comfortable post-program. After program completion, the average comfort range for all overlapping program learning objectives was between 4.4 and 5.6 for C1 and 4.4 and 5.7 for C2, with a maximum of very comfortable at 6. Retro-pre-self-assessments, conducted after program completion, largely recapitulated pre-program scoring, thus validating the participant’s sense of self-growth.

### Manager Assessments

Assessments were completed six months post-program completion by the managers of 26 participants from the first cohort of 32. Six missing assessments are due to staff turnover or no response. Data from C2 are not included, as they had not yet achieved 6 months post-program completion at the time of publication. Manager-scored assessments for C1 captured overall recruitment and retention competency levels for each participant before and six months after the program. As shown in Fig. 4 Panel A, growth in retention competency levels of C1 participants is apparent with an increase from 19% scored as Advanced pre-program to 50% scored Advanced post-program. Similar growth is shown for the overall recruitment competency with 31% scored as Advanced pre-program and 65% Advanced post-program.

**Fig. 4.**
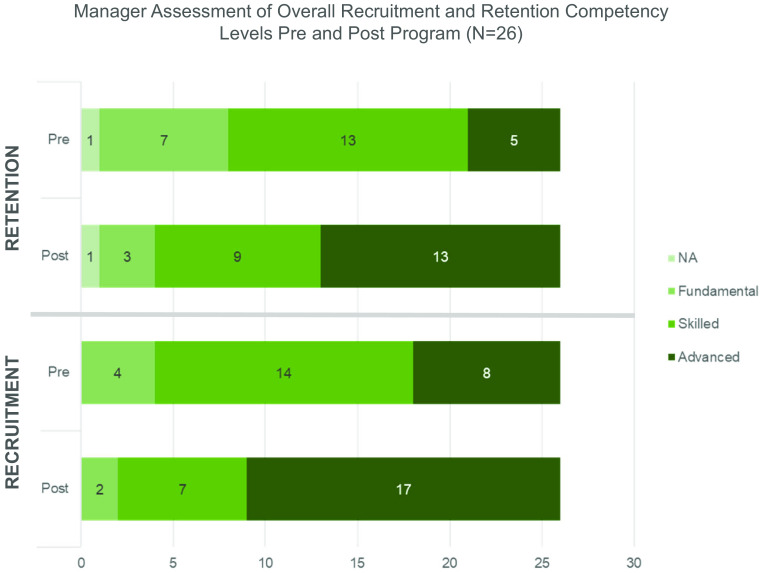
Panel A: The count of Clinical Research Professionals from Cohort 1 (*N* = 26 Total Participants) who achieved each competency level according to manager-scored assessments completed before and after program completion.

**Fig. 4.**
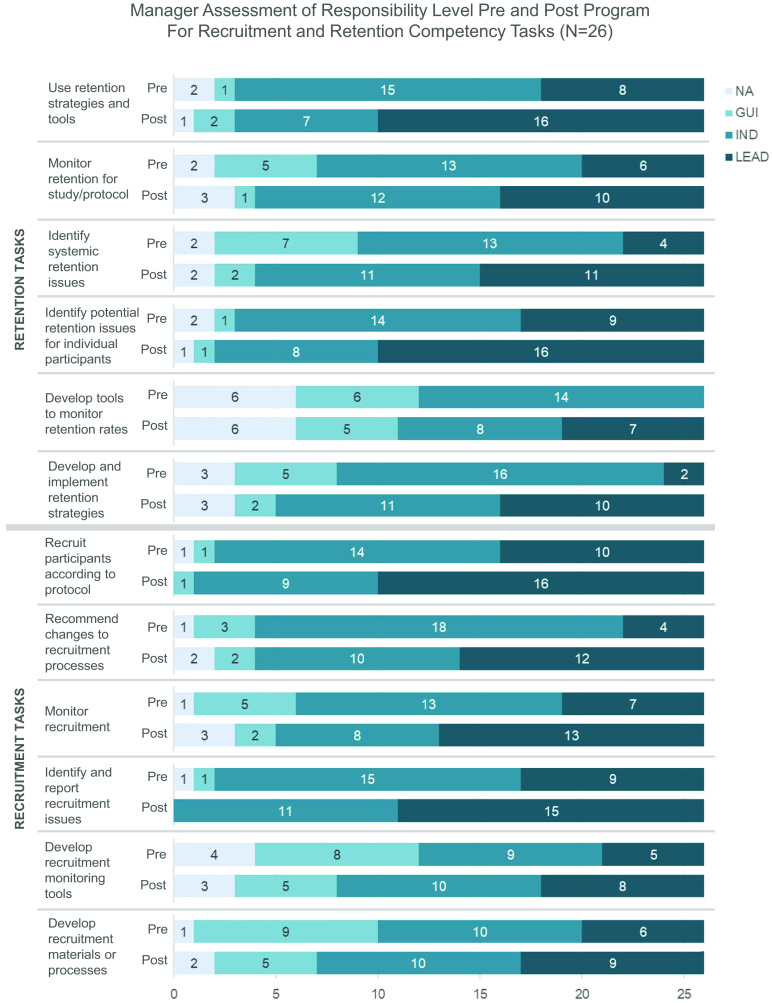
(Continued) Panel B: The count of Clinical Research Professionals from Cohort 1 (*N* = 26 Total Participants) that correspond to each manager-reported level of responsibility for recruitment and retention-related tasks before and after program completion: NA (not part of current job), GUI (does with guidance or assists), IND (does independently), LEAD (leads, trains, or mentors others in task).

The individual components comprising this manager-scored assessment show similar competency growth as shown in Fig. 4 Panel B. The highest level of responsibility growth was in “identifying systemic retention issues,” with 17 participants performing the task independently (13) or leading efforts (4) pre-program and 22 participants performing these tasks independently (11) or leading efforts (11) six months after program completion. Other notable growth occurred in “recommending changes to the recruitment process” (with 4 of 26 students leading the tasks pre-program and 12 leading post-program) and “identifying and reporting recruitment issues” (with 9 of 26 students leading pre-program and 15 leading post-program). Competence in each of the three tasks mentioned above is arguably critical for expanding staff confidence to identify current and future studies that could have more robust, EDI-focused recruitment and retention plans and to recommend more inclusive practices that they learned throughout the program. N/A responses within the manager assessment indicate that an individual does not perform the task as part of their current job. We suspect that growth of N/A responses from pre to post for some tasks indicates that individuals have changed roles or their managers had better understanding after program completion of what is involved in that task, and whether the employee performs it.

### Post-Class Satisfaction Surveys and Participant Interviews

Each class included a post-class satisfaction survey with responses used to iterate course objectives and content. For example, feedback received from participants in C1 necessitated changes to the structure and content covered in a few electives (See Table 1: E1, E2, E3, E4, E5, E6, E8).

Semi-structured interviews with 6 program participants contributed additional context around reasons for participation and impact of the program on daily work. An interview guide (Supplementary Materials F) focused on missing content, use and sharing of gained knowledge, barriers to incorporating learned concepts, and desired additional opportunities. We conducted an inductive thematic content analysis of the de-identified interview transcripts to identify common topics and themes across interviews. Following review of each transcript, the reviewers (JC and JR) catalogued summary topics mentioned by each interviewee and categorized them. As a final step, the categories were compared across interviews to identify themes and to ensure agreement between the two reviewers. The overarching themes that arose most frequently include the following:

1. EDI focus as a motivating factor to participate and key takeaway (6/6 interviewees – 21 mentions)
2. Appreciation and/or desire for more opportunities to connect and learn from the other CRPs enrolled in the program (6/6 interviewees – 16 mentions)
3. Application of the resources shared in the courses (6/6 interviewees – 15 mentions)
4. A desire for more “tips and tricks” and practical examples of how to implement strategies to improve ER&R efforts (6/6 interviewees – 12 mentions)
5. PI or unit buy-in and receiving a set protocol from the study sponsor as barriers to applying strategies learned (5/6 interviewees – 9 mentions)
6. General appreciation for the program itself (5/6 interviewees – 8 mentions)

Overall, results from the interviews and post-class evaluations indicated a positive experience, immediate application of many resources shared, and valuable takeaways from the perspective of the CRPs who completed the program. The interviewees provided instrumental feedback regarding future program improvements including the need to incorporate more time for sharing practical strategies, and providing learners with additional opportunities to connect with one another. Importantly, a few of our interviewees commented on confidence, confirming our hope that this program would increase their confidence in influencing ER&R efforts for the studies for which they are responsible. For example, interviewee 6 stated:

“This program really gave me the confidence to speak up and say ‘this is what has been successful for me,’ or you know ‘thanks for sharing that problem with the group, here’s resource that might be helpful’ or ‘this is what I would suggest,’ and so I think it just kind of gave me the voice to feel confident doing that. This just really gave me the confidence to speak up and have resources or evidence to back it up… I think other times, where I may have been quiet in meetings, now I’m like, actually, you know ‘here’s my idea and here’s where I got it from,’ and I feel very comfortable and confident doing that and I’m not sure if I would have without the program overall.”

Finally, it is notable that the most cited barriers to implementing what they learned are those around investigator buy-in and receiving sponsored protocols that are rigid and under-budgeted for proactive EDI, recruitment, and retention efforts.

## Discussion and Next Steps

Implementation of this program began with a draft list of topics to support skill development amongst clinical research staff as envisioned by one person (JR) based on years of experience working in the ER&R space. Turning that list into a viable curriculum required the effort and resourcefulness of a multidisciplinary team of individuals who appreciated the need and envisioned the value of such an endeavor. Identifying and engaging volunteer SMEs from across Duke was simple; however, meeting the demands of their personal calendars and schedules, especially as the world rolled into a full-blown pandemic, proved one of our greatest challenges. Designing a local curriculum with an expectation for eventual sharing with other research institutions represented a challenge. However, it also was an opportunity that led to identification and implementation of efficient tools, such as e-learning modules and facilitator guides.

Development of our program required significant personnel resources. These efforts were instrumental in creating a framework for the program that is available to other institutions by request. Over the first 2 years, the program development and implementation required approximately one full-time equivalent, split across roughly 3 to 4 primary personnel, plus volunteered effort from the 21 facilitators. Duke has a centrally funded clinical research support office [28] and CTSA-supported staff who were able to serve in program management and education coordinator roles to support building the program. Buy-in from CRPs, managers, and volunteer SMEs/facilitators across the institution who recognized the need and value of training was also an important component.

Session feedback permitted us to effectively evolve the program, resulting in greater self-perceived learning from C1 to C2. Courses where changes were made to the design showed greater average growth in comfort than those that did not include additional blended learning elements. Every course that added an additional e-learning module showed greater overall percentage increase in comfort for C2 over C1. The two courses with most notable growth for C2 over C1, *E3: Telling the Story of Your Research* and *E6: Remote Informed Consent*, may be attributed to changes made to the course materials, including incorporation of additional meaningful activities and practical examples, in response to participant feedback.

Both cohorts showed low initial comfort levels and high percentages of increase in the course objectives related to community and stakeholder engagement. Our hope is that significant growth in this area will embolden CRPs to form equitable and inclusive partnerships within our communities and to promote the benefit of such partnerships to their faculty investigators. Importantly, some of the strongest areas of average comfort growth were in EDI-related objectives. The courses with the least comfort growth on average due to high pre-program scores, *C7: Building Trust and Partnerships* and *C6: Active Listening to Enhance Respect and Awareness of Perspectives,* may reflect some degree of social desirability in the way people respond to their comfort with objectives such as *C6-O2: recognizing the importance of active listening* and *C7-O1: defining trust and trustworthiness*. Individuals may not wish to think of themselves as “bad listeners” or “untrustworthy.” What we do not assess, and perhaps should, is whether the pressure of the workday has an impact on CRPs’ active listening skills and reduces their ability to build trusting partnerships as a result.

Both the self-assessments of comfort and the manager-scored assessments of competency showed clear growth across ER&R-related objectives and tasks. The manager assessments completed for C1, in particular, show a clear indication of competency growth and an increase in ER&R task responsibility, including for those competencies that will hopefully bolster CRP confidence in identifying issues and recommending more inclusive practices for their studies. We recognize that managers were aware of their staff’s participation in the ER&R program which may confound post-program scoring. However, time between pre- and post-assessment lends confidence that these assessments reflect real behavior change.

Ultimately, the tangible measure of effectiveness of a program such as the ER&R Certificate will be an increase in participation and retention particularly among underrepresented populations. However, such measures require significant time post-intervention to realize. Moreover, confounding factors may make direct causal determinations difficult, such as additional enterprise-wide EDI initiatives that are being incorporated into the fabric of the institution, and the COVID-19 pandemic falling within the time span of the program. Much positive change is occurring across all Academic Medical Centers – with more intentional community engagement and unprecedented efforts to dismantle systemic racism and advance health equity in health care and research^2^ – thus, it is difficult to decipher what improvements are solely related to this specific effort. Our goal will be to continue to evaluate measures of inclusion and representation across Duke and especially related to programs impacted by our ER&R participants over the course of the next several years. Initiatives are already underway to better evaluate our enrollment and retention rates across all populations, data that will be made openly available to our community and ourselves so that we may hold ourselves accountable (and be held accountable by the community we serve) for improvements. This will enable us to look at the broad impact of all initiatives directed toward fostering more inclusive participation.

### Next Steps

We have invited at least one participant from another institution into each cohort, including individuals from UNC-CH and DTCC. Future iterations of the program will be offered in tandem with the DTCC Clinical Research Equity Scholars Program [29], so that students in the DTCC program may attend our courses as part of their experience. We see an opportunity to expand this collaboration to other institutions with similar programs to prepare participants for careers in clinical research.

The steering committee is licensing the program content repository to share with other institutions under a Creative Commons license, allowing them to custom tailor it to meet their needs and unique characteristics. Sharing this repository of work will allow other institutions to implement similar programs to benefit their CRPs without as much effort or cost. We are currently piloting this implementation initiative with colleagues at UNC-CH. We invite large academic health centers that implement similar programs to consider potential partnerships between institutions, like ours with DTCC, and to include local community clinics and other small local organizations where feasible. Collaborations like this will make training more accessible for clinical research programs in diverse healthcare settings and for individuals on clinical research career paths. Similarly, program content will be included in an e-library using Duke University’s Medical Center Library and Archives LibGuide resource. This will create an open-to-the-public searchable library of ER&R resources and tools provided within the program as well as additional resources as they are identified.

## Conclusion

Barriers to equitable ER&R exist at every level of clinical research participation opportunities, including those at the study design level (overly restrictive eligibility criteria and burdensome participation demands, etc.), system level (lack of flexible scheduling opportunities, resources and staffing, academic promotion and tenure practices that don’t acknowledge the real costs of managing research, stereotypes, biases, etc.), and community level (lack of knowledge, not being informed, distrust due to past atrocities, and lack of access). Each of these issues can and should be addressed both independently and in complementary ways, including educating investigators to mitigate barriers for which they have some measure of control (e.g. equitable recruitment practices, anti-bias and anti-racism, less-burdensome study designs, adequate ER&R funding or resources). According to program participants, some barriers to ER&R are built into the study protocols they are asked to implement. While this program iteration focused on staff development, as a next step we see value in using the ER&R program resources to design workshops for investigators to help them identify and mitigate these barriers before study protocols and budgets are finalized. The Just Ask content itself is a component of the recently released joint recommendations by the American Society of Clinical Oncology and Association of Community Cancer Centers for increasing racial and ethnic EDI in cancer clinical trials [30,31]. The Just Ask Training Program has already been widely adopted and used for 75 cancer clinical research sites nationally, including academic centers and community clinics, and was found effective in building awareness and skills toward addressing inequities in clinical research participation [30,31]. Additionally, sponsors and agencies such as the NIH and FDA are taking crucial steps toward addressing barriers through policy and guidance for clinical studies conducted in partnership with, or funded by, them [32,33].

With a baseline of trained staff and steady nomination numbers, the ER&R program will now be offered annually at Duke and also be available to students in CRP training programs at North Carolina Central University, UNC-CH, and DTCC. For the Duke CRPs who have completed the program, we are developing a train-the-trainer workshop that will allow them to use program materials to transfer the knowledge they have gained to colleagues in their units and serve as program facilitators if desired. Our hope is that this knowledge-sharing, along with the continually accessible wiki and e-library, will lead to more CRPs with awareness of the importance of inclusive and equitable recruitment to clinical research studies. To that end, we think it is important to continue constructing ways to build a community of participant-facing staff who are competent in ER&R practices and well-equipped to confidently champion inclusive practices in clinical research and trials. Results indicate that this program has been a step in the right direction toward expanding comfort and competence in these critically interrelated concepts for CRPs at Duke.

## Footnotes

1 The steering committee reviewed the post-class evaluation surveys for each class and made changes to course content and objectives to ensure that the entire program continues to evolve to meet the needs of the participants and respond to their feedback. Hence, objectives, content and course offerings have evolved.

2 To learn more about Duke’s efforts across the Health System and Schools of Medicine and Nursing, please visit the Duke Health Moments to Movement website.

## Supplementary material

For supplementary material accompanying this paper visit https://doi.org/10.1017/cts.2022.456. click here to view supplementary material
