# Supplementary material for: Development and evaluation of a novel training program to build study staff skills in equitable and inclusive engagement, recruitment, and retention of clinical research participants
Source: J Clin Transl Sci. 2022 Aug 30;6(1):e123. doi: 10.1017/cts.2022.456 (PMC9556271; doi:10.1017/cts.2022.456)
Supplement: Supplementary file 1 [file S2059866122004563sup001.zip › S2059866122004563sup004.docx]

**Cranfill et al. Supplemental Materials C**

**Sample Guidebook**

## ***Guidebook:* It’s Smarter to be Understood: Improving Readability in Participant Engagement Materials**

## This course is a guide to the basics of readability and tools to support the development of lay-friendly materials. We’ll discuss strategies you can use for your materials, from concise summaries to websites, flyers, and brochures.

| Pre-Learning | 1. Complete the required online module to prepare for discussion: Readability Fundamentals + Participant-Facing Engagement Materials. 2. Read article on translating evidence in uncertain times and come prepared to discuss your reaction. |
| --- | --- |
| Post Class Prompt | Take a moment to reflect on what you have learned in this course and in Nadine's Just Ask course. How can we use health literacy and understandable engagement materials to reach and be inclusive of all populations? |

## **Overview of Objectives and Activities:**

|  | **Learning Objectives** | **Core Activities** |
| --- | --- | --- |
| ***Online Pre-Class Content*** | | |
| **1** | Define readability and health literacy | Online Module: Lesson 1 – Intro to Health Literacy and Readability of Materials |
| **2** | Recognize the fundamentals of readability and how to use them to produce materials that participants can understand | Online Module: Lesson 2 – The Fundamentals of Readability and Lesson 3 – Applying the Fundamentals of Readability |
| **3** | Identify ways to assess readability and confirm understanding | Online Module: Lesson 4 – Assessing Readability and Confirming Understanding |
| ***In-Person Content*** | | |
| **1** | Define health literacy and associated challenges | Slide # 3 – Challenges  Slides # 4 to 8 – Defining health literacy |
| **2** | Recognize the importance of health literacy and readability in today’s scientific climate | Slides # 8 to 10 – bridging the gap and current events |
| **3** | Recall available tools for readability analysis and developing understandable materials | Slides # 11 – 15 – Briefly review tools available and see if they have ideas of others. |
| **4** | Apply readability foundations to produce materials potential participants can understand | Slides #16 – 19: Vincent’s PALM example  Slides # 21 – 22: Editing an Informed Consent Concise Summary  Slides #23-25: Jamie’s standard language update |
| **5** | Discuss health literacy and inclusivity strategies | Slide # 26 to End; review strategies |

## **Recommended Session for Smarter to be Understood**

**(20 minutes of online content + 1.5 to 2 hr in-person)**

ZOOM ADAPTATION: The team distributed handouts to participants in advance of the session via the course wiki. Participants will have any handouts on their devices to review or work with during the session. In place of a whiteboard or flip chart, the facilitators will use the chat and have students share their own screens during breakouts.

## **Introduction (2 mins)**

No matter how you are enrolling participants in a research study – in person or virtually, you will likely provide them with materials that convey information about that study and their potential or agreed participation in that study. These materials need to convey all the information they need to make an informed decision, and in a way that they are able to hear and understand. During this course we will walk through the foundations of health literacy, tools and basics of ensuring readability, and confirming understanding.

**SHOW:** Slide #1; briefly introduce the module (intro above) and provide an overview of the learning objectives for this module. ***Make sure to mention that they completed some content beforehand in the online module and we will ask them to share on what they learned.***

# **Objective 1: Define health literacy & associated challenges (10 mins)**

*Facilitators will introduce the challenges or the “why” they have found that it is important to discuss health literacy and readability. Then define health literacy using a visual example.*

**Topic #1:** **Challenges (5 minutes)**

**SHOW:** Slide #3 and walk through challenges that study staff often report.

**Topic #2: Define Health Literacy (5 minutes)**

**SHOW:** Slide #4
**SAY:** Definition (mention MRCT video from online module that defines health literacy) and share a practical example of health literacy. Renee’s cancer example illustrates that in times of crisis it is tough for anyone. ***OR ASK:*** *Based on the MRCT video you watched prior to class, how would you define Health Literacy and does anyone have an example you would be willing to share of how health literacy has impacted your own or someone else’s research interaction?*

**SHOW:** Slides #5-7

**SAY:** (5) Responsibility is ours for them to walk away with understanding. Here’s an example: My mother goes to her doctor’s office and her provider says: you need to lower your fat intake. Well, I don’t take in a lot of fat. While discussing diet she says “Well I drink two glasses of whole milk each day.” (6) Her provider says, that’s 16 grams of fat: That’s a lot! Okay? How much is it? (7) That equals 5 pieces of bacon every day. That’s health literacy!

**SHOW:** Slide #8

**SAY:** We want to avoid “I didn’t know” and “I didn’t understand.” These quotes came from two individuals who have different education levels and live in different places, but have had similar situations.

**Additional resources:** <https://www.healthypeople.gov/2020/tools-resources/evidence-based-resource/national-action-plan-improve-health-literacy>

# **Objective 2: Recognize the importance of health literacy and readability in today’s scientific climate (15 mins)**

**Topic 1: Bridging the Health Literacy Gap (5 min)**

**SHOW:** Slide #9
**SAY:** Why is readability so important? About a third to half the U.S. population reads at or below at or below an 8th grade level. People who have chronic diseases – among other vulnerable groups– are more likely to have a lower reading grade level. And yet, information about science in general, including medicine, is becoming increasingly less readable. Readability across many disciplines is decreasing over time, most likely because of increasing specialization and its associated jargon. The better and more sophisticated science is becoming, the further away it is drifting from being understandable by the public and the communities that could use the information.

**Topic 2: Current/Relevant Events and Literacy (10 mins)**

**SHOW:** Slide #10 - Article;

**ACTIVITY #1:** Impacts of literacy and unintentional misinformation. Have 2 people (at most) from class share brief reactions from article *Beyond Evidence Reporting: Evidence Translation in an Era of Uncertainty.*

**SAY:** facilitator debrief responses and share own takeaways.

**SHOW:** Slide #10 – Website and Flyers;

**SAY:** This makes it all the more important to be clear and provide materials that clearly answer questions and present facts in a way that make them understandable. The ACTIV-2 study and MRCT COVID flyers are a great example of this and they are linked in the slides.

# **Objective 3: Recall available tools for readability analysis and developing understandable materials (3 mins)**

**Topic 1:** Slides #12-15. Readability tools and strengths and weaknesses. (3 Minutes quick review, all of this is in the pre-work online module)

**SHOW:** Slide 12
**SAY**: *Disclaimer:* Someone who produces materials for recruitment and participants, will need to learn how to produce readable content without always relying on readability tools (which can have blind spots), but they are a good place to start for learning how to make a move toward readability.

**SHOW:** Slides 13-15
**SAY**: You reviewed these tools in the online module and we will provide links to them again in the slides.
**ASK on Slide 15:** Do any additional tools come to mind that you use often and have been helpful? *Share in the chat and people can take the time to check them out if they’d like to after the session.* ***We encourage you to share your examples/ideas of tools in the Wiki for others to benefit from after the session.***

# **Objective 4: Apply readability foundations to produce materials potential participants can understand (40 min)**

**Topic 1: Slides #16-19. Vincent’s PALM study example (15 Minutes)**

**SHOW:** Slides 17-19
**SAY/Adaptation for other institutions**: Share example of a study where this was done well and the facilitator is proud of the result. This is what we wanted to accomplish, these were the goals we set, this is how we got there. (e.g. Vincent’s PALM example reduced consent document into a 6-minute video to show in waiting room).

**Topic 2: Concise Summary Activity: Slides 20 – 22 (20 min)**

**SHOW**: Slide 20 – poorly written summary and statistics
**SAY:** Here is the poorly written concise summary that you reviewed as part of your pre work.

**SHOW**: Slide 21
**SAY:** Provide instructions for breakout activity.
**Activity**: In breakout groups, students should recall their review the summary, discuss in their groups and highlight where they found issues, and note improvements that could be made to the layout. (10 min) *When we come back together, rather than having everyone share, ask* ***ONE*** *group to volunteer to share. Then ask if anyone has anything else/different to add. Briefly summarize as appropriate and move on. (5 min)*

**SHOW:** Slide 22 – Show improved concise summary
**SAY:** point out any highlighted items that they didn’t mention. (5 min)

**Topic 3: Standard Language Terms (3 minutes)**

**SHOW:** Slide 23 – 25
**SAY:** The Duke RIC is working with the IRB to make edits to the Standard Language they have out there for studies. Briefly show the examples.
**Adaptation for other institution:** Share information about standard language templates at your institution – where to find them and how to use them. If there are issues with the language, be open about them.

# **Objective 5: Discuss health literacy and inclusivity strategies (25 min)**

**Topic 1: Health Literacy Strategies – (15 min)**

**SHOW:** Slide 26 – Health Literacy Strategies – 5 min
**SAY:** In the online module you learned about different strategies for improving readability and assessing health literacy. During this speed round we will throw up a topic and you type in the chat what stood out to you that you plan to start applying right away about each – broad or specific.
**ACTIVITY (5 min):** Click to bring up the topic for 45 seconds at most - and read out/reinforce responses from students as they come into the chat.

**SHOW:** Slide 27– Assessing Readability – 3 min **SAY:** You learned about assessing understanding in the online module. But just to reinforce here – ask people to look over the content and test it prior to using it. Test with your actual audience and at the very least – read it out loud! Is that how you would say it to someone sitting next to you, engaged in conversation with you?

Anticipate questions that might come up if someone has all the freedom, safety, and space to question every word.

**SHOW:** Slide 28– Confirm Understanding (reinforce – this is in module) – 7 min
**SAY:** the teach-back method is where you ask them to tell you what they understood about the information given to them. You are explaining, assessing, clarifying, and measuring understanding all in one interaction. If you find something needs to be changed – change it (amend it and submit to the IRB) -- this is the right thing to do if you notice a lack of understanding about a certain section or concept.
**ASK:** what other types of questions can you ask to confirm understanding during teach back method?

**Topic 2: Inclusivity Strategies – (10 min)**

**SHOW:** Slide 29

**SAY:** these are some tips for how to be inclusive when developing materials – tell them what is on the slide.

**SHOW:** Slide 30  **SAY:** These quotes illustrate why it is important to avoid stereotypes and bias – and be aware of needs and accommodations for certain conditions. Think about whether there are stigmas associated with the condition you are studying and how to communicate about that condition considering any associated stigmas.

# **Conclusion (3 min) Slide #31 – This is bad enough video**

“In this course, we’ve spent time talking about the importance of health literacy and our responsibility for getting to a place where our participant understand the materials we share with them. We encourage you to consider ways, and possibly think outside the box, for ways to make sure understanding is achieved for your studies. We’d like to close with this video: “This is bad enough” was written by poet Elspeth Murray for the launch of the cancer information reference group of SCAN, the South East Scotland Cancer Network

<https://www.youtube.com/watch?v=R3tJ-MXqPmk>

Use any additional time for discussion and questions.

Thank you.
