# Supplementary material for: Development and evaluation of a novel training program to build study staff skills in equitable and inclusive engagement, recruitment, and retention of clinical research participants
Source: J Clin Transl Sci. 2022 Aug 30;6(1):e123. doi: 10.1017/cts.2022.456 (PMC9556271; doi:10.1017/cts.2022.456)
Supplement: Supplementary file 1 [file S2059866122004563sup001.zip › S2059866122004563sup001.docx]

**Cranfill et al. Supplemental Materials A**

**2022 Duke ER&R Program Course Objectives**

| **Core Courses (Participants Complete All)** | | |
| --- | --- | --- |
| **Course** | | **Objectives** |
| Just Ask: Equity and Diversity in Clinical Research  *Online Pre-Learning Module: Just Ask: Intro to Equity and Diversity in Clinical Research* | - Define health disparity - Define health equity - Discuss what keeps diverse populations from accessing clinical research opportunities - Describe your role in promoting diversity in clinical research - Identify your own implicit biases - Recognize how bias impacts recruitment and engagement in clinical research | |
| Clinical Research Recruitment, Regulations, Best Practices, and Tools***** | - Discuss Duke policies related to recruitment and engagement - Recognize the importance of planning for recruitment - Recognize the process for obtaining IRB approval for recruitment plans and materials - Recall Duke Health branding requirements for materials and ads - Use Maestro Care tools to identify eligible participants | |
| Smarter to be Understood: Improving Readability  *Online Pre-Learning Module: Readability Fundamentals + Participant Facing Engagement Materials* | - Define readability and health literacy - Apply readability foundations to produce materials potential participants can understand - Identify ways to assess readability and confirm understanding - Recognize the importance of health literacy, readability and inclusivity in today’s scientific climate - Use available tools to perform a readability analysis of engagement materials - Recognize tools at Duke to develop lay-friendly materials | |
| Active Listening to Enhance Respect and Awareness of Participant Perspectives | - Define active listening - Recognize the importance of active listening and how it can lead to both respectful and aware engagement and recruitment practices - Identify strategies to build your capacity for hearing and respecting others - Recognize why listening is an important patient-centered engagement approach - Identify ways to shift your lens and consider other perspectives | |
| Using Social Marketing Principles to Design Your Engagement Strategy | - Define social marketing - Describe how evidence-based social marketing can be used to develop engagement and recruitment materials and strategies - Identify strategies to recognize your audience and recruitment strategies that will resonate with them - Discuss how formative research can help reach your audience - Describe the importance of tracking implementation and assessing outcomes of a recruitment strategy | |
| Building Trust and Partnerships | - Define trust and trustworthiness - Recognize the importance of trust between study team and participants - Discuss strategies for ensuring positive research interactions - Identify strategies for building trust with the community at large | |
| Retention: Challenges and Opportunities  *Online Pre-Learning Module: Strategies to Support Retention of Clinical Research Participants* | - Describe the importance of strong retention practices - Set study expectations and explain them clearly to support retention - Discuss strategies for relationship-building to support retention - Identify ways to assess continual participant interest in a study - Recognize ways to discuss the importance of study continuation without coercion | |

| **Elective Courses (Participants Choose 3)** | |
| --- | --- |
| **Course** | **Objectives** |
| Principles and Best Practices of Stakeholder Engagement  *Online Pre-Learning Module: Community & Stakeholder Engagement* | - Recognize fundamental principles and practices for clinical research stakeholder engagement - Identify various stakeholder engagement strategies for clinical research - Describe how to identify stakeholders for a given study - Discuss stakeholder engagement in the design and implementation of engagement, recruitment and retention strategies and materials - Describe Duke tools and resources to help you identify and engage with stakeholders |
| Social Media: Is it right for your research?  *Online Pre-Learning Module: Using Social Media for Study Recruitment: Questions to Consider* | - Describe how social media is used for recruitment in research studies - Identify questions to determine whether social media is right for a study - Recognize different platforms and the benefits of using them as a recruitment option - Discuss how social media ad campaigns work and how to leverage existing Duke channels - Recognize the components of a social media marketing plan - Find Duke guidelines, policies, procedures, and resources for marketing studies on social media |
| Community Engaged Research Initiatives (CEnR)******  *Online Pre-Learning Module: Community & Stakeholder Engagement* | - Define Community, Community Engagement, and Community Engaged Research (CEnR) - Describe the Principles of CEnR - Describe why CEnR is important to addressing local priorities and improving well-being - Describe how CEnR can address trust, increase diversity and inclusiveness, and improve equity - Discuss the spectrum of community engagement in research - Find tools and resources to help you engage with the community |
| Telling The Story of Your Research | - Recognize opportunities to tell a story throughout different stages of a study - Recognize ways to communicate about research to a variety of audiences - Discuss strategies for making research inviting rather than overwhelming - Identify tools to develop lay summaries of study results |
| Engagement, Recruitment, and Retention on a Shoestring | - Identify and plan for the real costs of recruitment and retention - Recognize ways to plan for recruitment and engagement with a limited budget - Find tools and strategies for using your available recruitment budget |
| Remote Informed Consent: Design and Delivery Practices  *Online Pre-Learning Module: Elements of Effective eConsent Design* | - Recognize elements of effective eConsent design (cognitive load, multimedia, and interactivity) - Discuss components of user-centered design - Discuss social and cultural dynamics that are known to affect participant attitudes and preferences with respect to remote consent delivery - Identify ways to effectively use REDCap to create an engaging, informative eConsent |
| 5Ts: A Framework to Support Inclusion of Older Adults in Research | - Discuss case examples related to recruiting older adults - Recognize why it is difficult to recruit older adults and why they are a special population - Define a framework for supporting inclusion of older adults in research - Identify ways to include older adults in your studies |
