# Supplementary material for: Development and evaluation of a novel training program to build study staff skills in equitable and inclusive engagement, recruitment, and retention of clinical research participants
Source: J Clin Transl Sci. 2022 Aug 30;6(1):e123. doi: 10.1017/cts.2022.456 (PMC9556271; doi:10.1017/cts.2022.456)
Supplement: Supplementary file 1 [file S2059866122004563sup001.zip › S2059866122004563sup002.docx]

**Cranfill et al. Supplemental Materials F**

**Engagement, Recruitment, and Retention Certificate Program**

**Semi-structured Interview Guide**

You were a student in one of the initial cohorts of the Engagement, Recruitment and Retention Certificate Program. We are interested in learning more about your experiences with the program and the impact you feel it has (or will have) on your work. To that end, we are conducting semi-structured interviews with some of the students.

1. Our first questions are regarding how and why you chose to join the program and what you hoped to learn.
   1. Did you suggest the opportunity to participate in the program to your nominator or did your nominator suggest it to you?
      1. PROBE: Tell me more about that.
      2. PROBE: What did you hope to learn?

PROBE: Tell me more about that

1. In your cohort, there were XX Core Courses that had to be taken (share list on Zoom or ahead of time):

- *Just Ask: Equity and Diversity in Clinical Research*
- *Recruitment Regulations, Tools and Best Practices*
- *It’s Smarter to Be Understood: Plain Language and Readability Principles*
- *Retention Challenges and Opportunities*
- *Social Marketing Principles*
- *Respect and Awareness for Participant Perspectives*
- *Building Trust and Partnerships (changed from elective to core for Cohort 2)*
  1. What did you think of the Core Courses?
     1. PROBE: Was there anything missing that we should consider including as a core course for future cohorts?
        1. PROBE: Tell me more about that
     2. Were there any courses you thought should not be included as core courses?
        1. PROBE: Tell me more about that

1. In your cohort, there were XX Electives from which you had to select at least 2 (or 3 for C2) [share list on Zoom or ahead of time]

- *Social Media Marketing for Clinical Research*
- *Telling the Story of Your Research*
- *Community, Patient and Stakeholder Engagement (Cohort 1)*
  - *Community Engaged Research Initiatives (Cohort 2)*
  - *Stakeholder Engagement Principles and Strategies (Cohort 2)*
- *Budgeting for Engagement and Recruitment on a Shoestring*
- *Remote Informed Consent Design and Delivery*
- *5Ts for Engaging Older Adults in Research*
  1. What did you think of the Elective Courses?
     1. PROBE: Was there anything missing that we should consider including as an elective for future cohorts? Were there any electives that we should consider changing to a Core Course?
        1. PROBE: Tell me more about that

1. One of the goals of the certificate program is to build the skills of study staff who can return to their units to serve as an engagement and recruitment resource/champion/mentor for their colleagues. Have you had any opportunities to share what you learned during the program with people in your unit?
   1. PROBE: Tell me more about that.
   2. PROBE: How were those sessions structured? (1 on 1 conversation, sharing at a meeting, etc.)
   3. PROBE: Would you recommend the program attendance to someone else in your unit?
2. We’d like to know more about how you’re applying what you learned to your work. Could you share an example of an opportunity you’ve had to use what you learned in your daily work?
   1. PROBE: Tell me more about that
3. What kinds of barriers or challenges have you encountered in the application of what you learned to your work?
   1. PROBE: Tell me more about that
      1. PROBE: Are there things your department or unit should be doing to alleviate those barriers or challenges?
         1. PROBE: Tell me more about that
      2. PROBE: Are there things the institution should consider doing to alleviate those barriers or challenges?
         1. PROBE: Tell me more about that
4. What opportunities would you like to have to ***apply*** what you learned in the program?
   1. PROBE: Tell me more about that
5. Finally, are there any additional opportunities you would like to have as a result of having participated in this program? For instance, serving on committees or groups working to make Duke Research more participant-centered, speaking-engagements, tier advancement and professional growth, etc.
   1. PROBE: Tell me more about that
