# Supplementary material for: Development and evaluation of a novel training program to build study staff skills in equitable and inclusive engagement, recruitment, and retention of clinical research participants
Source: J Clin Transl Sci. 2022 Aug 30;6(1):e123. doi: 10.1017/cts.2022.456 (PMC9556271; doi:10.1017/cts.2022.456)
Supplement: Supplementary file 1 [file S2059866122004563sup001.zip › S2059866122004563sup003.docx]

**Cranfill et al. Supplemental Materials G**

**Data Tables for Self-Assessments of Comfort and Manager-Score Assessments**

Link to Excel File in Duke Box: <https://duke.box.com/s/1l6fqc8wok0e7gkkhujea7868jyg5y3y>
