# Supplementary material for: Development and evaluation of a novel training program to build study staff skills in equitable and inclusive engagement, recruitment, and retention of clinical research participants
Source: J Clin Transl Sci. 2022 Aug 30;6(1):e123. doi: 10.1017/cts.2022.456 (PMC9556271; doi:10.1017/cts.2022.456)
Supplement: Supplementary file 1 [file S2059866122004563sup001.zip › S2059866122004563sup006.docx]

**Cranfill et al. Supplemental Materials E**

**Manager Assessment of Retention Competency/Task Responsibility**

#### RESEARCH OPERATIONS: PARTICIPANT RETENTION Candidate name: Click or tap here to enter text.

#### Manager-Scored Assessment Date: Click or tap here to enter text.

| You are welcome to discuss with the employee prior to or during scoring, as you are attesting to their level of responsibility with each task. Please complete this document in its entirety before returning it to the program team. The competency table below is an overview of what an employee should be doing at the Fundamental, Skilled, and Advanced levels. Completion of the table below will help you confirm your employee’s competency level. |
| --- |

| **COMPETENCY:** Employ strategies to maintain retention rates. Evaluate processes to identify problems with retention. May train or oversee others. | **FUN** | **SKI** | **ADV** |
| --- | --- | --- | --- |
| - Implement retention strategies. Identifies potential retention issues for individuals and escalates appropriately. | 🗹 | 🗹 | 🗹 |
| - Recognizes systematic retention issues, and develops and implements retention strategies. Monitors retention rates. |  | 🗹 | 🗹 |
| - Develop tools for monitoring retention rates or help other teams with retention strategies. Mentor and train others to follow retention strategies and to identify potential issues. |  |  | 🗹 |

**MANAGER ASSESSMENT OF EMPLOYEE**

1. Think about your employee’s role with regard to recruitment and describe their highest level of responsibility for the following tasks.
   - GUI (do with guidance or assist with task)
   - IND (do task independently)
   - LEAD (lead, train, or mentor others in the task)
   - NA (not applicable/not part of their job)

| Task |  | Level of responsibility | | | |
| --- | --- | --- | --- | --- | --- |
|  |  | GUI | IND | LEAD | NA |
| 1. Use retention strategies and tools |  |  |  |  |  |
| 1. Identify potential retention issues for individual research participants |  |  |  |  |  |
| 1. Identify systematic retention issues |  |  |  |  |  |
| 1. Monitor retention for entire study/protocol |  |  |  |  |  |
| 1. Develop and implement retention strategies |  |  |  |  |  |
| 1. Develop tools to monitor retention rates |  |  |  |  |  |

To meet **FUNDAMENTAL**, candidate must meet at least all items marked in blue boxes.

To meet **SKILLED**, candidate must meet at least the **FUNDAMENTAL** level, and at least the items marked in the orange boxes.

To meet **ADVANCED**, candidate must meet at least the **SKILLED** level, and at least the items marked in the green boxes.

| **RESULTS OF ASSESSMENT** |
| --- |
|  |
| **Competency level achieved:**   Fundamental  Skilled  Advanced  Does Not Meet Fundamental |
|  |
| **Rater/scorer name:**  Click or tap here to enter text. |
|  |
| **Additional comments if desired:** Click or tap here to enter text. |
