# Supplementary material for: Development and evaluation of a novel training program to build study staff skills in equitable and inclusive engagement, recruitment, and retention of clinical research participants
Source: J Clin Transl Sci. 2022 Aug 30;6(1):e123. doi: 10.1017/cts.2022.456 (PMC9556271; doi:10.1017/cts.2022.456)
Supplement: Supplementary file 1 [file S2059866122004563sup001.zip › S2059866122004563sup007.docx]

**Cranfill et al. Supplemental Materials D**

**Manager Assessment of Recruitment Competency/Task Responsibility**

#### RESEARCH OPERATIONS: RECRUITMENT Name: Click or tap here to enter text.

#### Manager-Scored Assessment Date: Click or tap here to enter text.

| You are welcome to have a conversation with the employee prior to or during scoring, as you are attesting to their level of responsibility with each task. Please complete this document in its entirety before returning it to the program management team. Be sure to answer all questions required for the competency level that you believe the employee has reached. The competency table below is an overview of what an employee should be doing at the Fundamental, Skilled, and Advanced levels. The questions in Part 1 and 2 will help you confirm your employee’s competency level. |
| --- |

| **COMPETENCY:** Employs and may develop strategies to maintain recruitment rates and evaluate processes to identify problems. Escalates issues. May train or oversee others. | **FUN** | **SKI** | **ADV** |
| --- | --- | --- | --- |
| - Follows the recruitment process defined by the protocol, SOP, or other study documentation; identifies and reports potential issues with recruitment. Knows and works towards a recruiting target or goal. | 🗹 | 🗹 | 🗹 |
| - Assists with or independently develops study recruitment processes or documents. Provides solution(s) for recruitment challenges. |  | 🗹 | 🗹 |
| - Monitors recruitment, develops recruitment processes/docs and monitoring tools, and recommends changes to recruitment processes. Shares expertise in recruitment with other study teams. |  |  | 🗹 |

**MANAGER ASSESSMENT OF EMPLOYEE PART 1: Level of Responsibility for Tasks**

1. Think about your employee’s role with regard to recruitment and describe their highest level of responsibility for the following tasks.
   - GUI (do with guidance or assist with task)
   - IND (do task independently)
   - LEAD (lead, train, or mentor others in the task)
   - NA (not applicable/not part of their job)

| Task |  | Level of responsibility | | | |
| --- | --- | --- | --- | --- | --- |
|  |  | GUI | IND | LEAD | NA |
| 1. Recruit participants according to protocol |  |  |  |  |  |
| 1. Identify and reports potential issues |  |  |  |  |  |
| 1. Develop recruitment materials or processes |  |  |  |  |  |
| 1. Recommend changes to recruitment processes |  |  |  |  |  |
| 1. Monitor recruitment |  |  |  |  |  |
| 1. Develop recruitment monitoring tools |  |  |  |  |  |

**MANAGER ASSESSMENT PART 2**

| **You must select either Yes or No in checkboxes below.** |
| --- |

To meet **FUNDAMENTAL**, meet at least the items marked in the blue boxes and all items marked below.

| Is responsible for recruiting participants independently | Yes /  No |
| --- | --- |
| Demonstrates understanding of tools associated with recruitment process for this protocol | Yes /  No |
| Able to identify and report recruitment issues | Yes /  No |
| Knows expected recruitment rates, and tools and strategies needed to monitor and maintain | Yes /  No |
| From your experience, demonstrates a basic understanding of:   - - **Ethical human subject recruitment practices**   - **Clinical research recruitment regulations**   - Culturally appropriate mechanisms for recruiting | Yes /  No |

*If you checked the blue boxes in the table above and marked all of the checkboxes above with “yes,” then this employee meets* ***FUNDAMENTAL*** *competency for recruitment.*

To meet **SKILLED**, candidate must meet at least the **FUNDAMENTAL** level, at least the items marked in the orange boxes, and all items below.

| Develops recruitment processes and/or tools in accordance with institutional standards  When faced with recruitment issues, candidate recommends potential solutions | Yes /  No |
| --- | --- |
| From your experience, demonstrates a strong understanding of, and implements:   - - **Ethical human subject recruitment practices**   - **Clinical research recruitment regulations**   - Culturally appropriate mechanisms for recruiting | Yes /  No |

*If you checked all the orange and blue boxes in the table above and marked all of the checkboxes above with “yes,” then this employee meets* ***SKILLED*** *competency for recruitment.*

To meet **ADVANCED**, candidate must meet at least the **SKILLED** level, at least the items marked in the green boxes, and all items below.

| Leads the implementation of changes to recruitment processes | Yes /  No |
| --- | --- |
| Shares expertise in recruitment with other study teams | Yes /  No |

*If you checked all the green, orange, and blue boxes under the “IND” column in the table above and marked all of the checkboxes above with “yes,” then this employee meets* ***ADVANCED*** *competency for recruitment.*

| **RESULTS OF ASSESSMENT** |
| --- |
|  |
| **Competency level achieved:**   Fundamental  Skilled  Advanced  Does Not Meet Fundamental |
|  |
| **Rater/scorer name:**  Click or tap here to enter text. |
|  |
| **Additional comments if desired:** Click or tap here to enter text. |
