# Supplementary material for: Development and evaluation of a novel training program to build study staff skills in equitable and inclusive engagement, recruitment, and retention of clinical research participants
Source: J Clin Transl Sci. 2022 Aug 30;6(1):e123. doi: 10.1017/cts.2022.456 (PMC9556271; doi:10.1017/cts.2022.456)
Supplement: Supplementary file 1 [file S2059866122004563sup001.zip › S2059866122004563sup005.docx]

**Cranfill et al. Supplemental Materials B**

**Sample Learning Plans**

# ER&R Curriculum Learning Plan Sample 1

Course content Due Date – mm/dd/yyyy

Course Title: *It’s Smarter to be Understood: Improving Readability in Participant Engagement Materials*

***Related Competency Domains:***

- *Safety and Ethics: Development of Informed Consent Doc and Plan*
- *Research Operations: Recruitment*

| **Duke Content SME/Facilitator** | Jamie Roberts, Renee Leverty, Vincent Miller |
| --- | --- |
| **Method: Blended** | Online Module followed by 120 min lecture and interactive workshop |
| **Pre-Learning** | 1. Complete the online module to prepare for discussion:**Readability Fundamentals + Participant-Facing Engagement Materials.** 2. Read **article** on translating evidence in uncertain times and come prepared to discuss your reaction. |
| **Description** | A guide to health literacy and readability when creating participant engagement materials. Participants will learn strategies to support and confirm participant understanding of study materials. We’ll discuss tools you can use to design your materials (from consent forms and concise summaries to websites, flyers and brochures) to be more inclusive and readable by all potential participants. |
| **Learning Objectives** | Online Module:   1. Define readability and health literacy 2. Recognize the fundamentals of readability and how to use them to produce materials that participants can understand 3. Identify ways to assess readability and confirm understanding   Face to Face:   1. Define health literacy and associated challenges 2. Recognize the importance of health literacy and readability in today’s scientific climate 3. Recall available tools for readability analysis and developing understandable materials 4. Apply readability foundations to produce materials potential participants can understand 5. Discuss health literacy and inclusivity strategies |
| **Journaling Prompt** | Take a moment to reflect on what you've learned in this course. How can we use health literacy and understandable engagement materials to reach and be inclusive of all populations? OR Describe how developing materials that participants understand supports opportunities for ***everyone*** to participate in research. |
| **Resources** | List of resources for Course Wiki included here. |

# Course Outline Tied to Learning Objectives

## Define health literacy and associated challenges

- List content and **activities** to meet this objective.

## Recognize the importance of health literacy and readability in today’s scientific climate

- List content and **activities** to meet this objective.

## Recall available tools for readability analysis and developing understandable materials

- List content and **activities** to meet this objective.

## Apply readability foundations to produce materials potential participants can understand

- List content and **activities** to meet this objective.

## Discuss health literacy and inclusivity strategies

- List content and **activities** to meet this objective.

# ER&R Curriculum Learning Plan Sample 2

Course content Due Date – mm/dd/yyyy

Course Title: *Building Trust and Partnerships in Clinical Research*

***Related Competency Domains:***

- *Research Operations: Recruitment*
- *Research Operations: Participant Retention*
- *Research Operations: Study Visits*

| **Duke Content SME/Facilitator** | Dr. Leonor Corsino, Sabrena Mervin-Blake, Raquel Ruiz, Jaimie Roberts |
| --- | --- |
| **Method: Blended** | Pre-Learning Videos followed by 30min Discussion, 60min Panel, and 30min Q&A |
| **Pre-Learning** | 1. Watch Frances Frei: [How to Build (and Rebuild) Trust Video](https://www.ted.com/talks/frances_frei_how_to_build_and_rebuild_trust?language=en) (Runtime: 14min) 2. Watch Onora O’Neill (min 4:43 – end): [What We Don’t Understand About Trust](https://youtu.be/1PNX6M_dVsk?t=283) (Runtime: 5min) 3. Watch the AAMC [Principles of Trustworthiness Community Video](https://www.aamc.org/trustworthiness#video) (Runtime: 11min) and review the accompanying [Principles of Trustworthiness guide](https://www.aamchealthjustice.org/media/271/download?attachment). |
| **Description** | A dive into the importance of trust and trustworthiness to the partnerships between study team and participant and researchers and the community at large. We will discuss these concepts along with strategies for ensuring positive research interactions with diverse communities that go beyond individual study participation and create clinical research champions across the community. |
| **Learning Objectives** | Upon completion of the course, you will be able to:   1. Define Trust, Trustworthiness, and Vulnerability 2. Recognize the importance of building trust and being trustworthy 3. Discuss strategies for building trust and ensuring positive research interactions with various communities of participants |
| **Journaling Prompt** | After watching the AAMC Center for Health Justice’s Principles of Trustworthiness video and reviewing the 10 Principles Guide, which principle stood out to you as one you would most like to implement in the studies you are involved in and how could it help you to equitably partner with the communities you engage? |
| **Resources** | List of resources for Course Wiki included here. |

# Course Outline Tied to Learning Objectives

## Define Trust, Trustworthiness, and Vulnerability

- List content and **activities** to meet this objective.

## Recognize the importance of building trust and being trustworthy

- List content and **activities** to meet this objective.

Discuss strategies for building trust and ensuring positive research interactions with various communities of participants

- List content and **activities** to meet this objective.
